# Calcification patterns of a foundation species reveal resilience to environmental change in southern California over the twentieth century

**DOI:** 10.1098/rspb.2026.1964

**Published:** 2026-10-21

**Authors:** Veronica P. Vriesman, Esther Kennedy, Gabriela Van Ausdale, Sandra Carlson, Tessa M. Hill

**Affiliations:** ^1^ Department of Natural Sciences, Scripps College, Claremont, CA, USA; ^2^ Department of Natural Sciences, Pitzer College, Claremont, CA, USA; ^3^ Alaska Coastal Rainforest Center, University of Alaska Southeast, Juneau, AK, USA; ^4^ Earth and Planetary Sciences, University of California Davis, Davis, CA, USA; ^5^ Bodega Marine Laboratory, University of California, Davis, Bodega Bay, CA, USA

**Keywords:** calcification, climate change, California Current System, ocean warming, ocean acidification, California mussels, molluscs, bivalves, biomineralization

## Abstract

Oceanographic changes due to carbon dioxide emissions have complex and varied impacts on marine calcifying taxa. Changes in calcification can unfold over decades or longer, requiring a longer-term understanding of biomineralization patterns than laboratory-based studies can provide. Here, we investigate the calcification history of the marine mussel *Mytilus californianus* to determine how this ecologically critical foundation species has responded to past and ongoing environmental changes in the southern end of its range within the California Current System (CCS) between 27.72° N and 34.47° N. Calcification metrics based on shell morphology, growth banding, and mineralogical structure in 130 museum-archived *M. californianus* specimens collected decadally from 1913 to 2010 revealed unexpected resilience in southern *M. californianus* shells after previous studies found calcification vulnerability in this species farther north. Shell growth data were paired with temperature datasets and regional pH records. Despite increasingly warmer and more variable conditions in the southern CCS, *M. californianus* shell features remained quantitatively unchanged in this region since 1913, although we present preliminary microstructural data that align with previous evidence of increased crystallographic disorder in this species. Based on overall consistencies in calcification metrics, *M. californianus* appears more resistant to environmental changes at the southern end of its range.

## 1. Introduction

Anthropogenic carbon dioxide emissions are causing significant changes to global oceans [1,2]. Multiple meta-analyses and experimental observations have found that elevated temperatures and *p*CO_2_ levels negatively impact marine calcifying taxa by disrupting metabolic processes, altering energy budgets, and reducing calcification rates [3–7]. Calcification shifts have the potential to significantly alter ecosystem functioning [6,8,9] when ecologically important species are adversely affected [3]. Given that biotic responses to ocean acidification (OA) and sea surface temperature (SST) changes can be highly varied [10], it is difficult to quantify the impacts of a changing ocean on marine calcifying taxa [5,11,12]. While experimental work is useful for documenting the immediate impacts of acute conditions, a long-term record of calcification is vital for accurately characterizing responses unfolding over multiple years [13]. To better understand the history of biological responses to changing climate conditions and establish calcification baselines, some studies have used archival collections of biogenic carbonates from the past century or longer to identify and contextualize changes in bivalve shell characteristics, including shell morphology, mineralogy and microstructure [14–18].

Archived shells supplement the information gleaned from field monitoring studies and experimental observations by contextualizing biotic changes over timescales relevant to ecological and evolutionary processes. One species of particular ecological significance is *Mytilus californianus*, a marine mussel that dominates rocky intertidal environments from the Aleutian Islands, Alaska, USA to Baja California Sur, Mexico [19]. *Mytilus californianus* provides a habitat for hundreds of encrusting species [19–21] and is therefore a foundation species for the northeastern Pacific coastline [22]. Changes in *M. californianus* calcification patterns could have ecosystem-wide impacts if *M. californianus* shells become thinner or weaker, and therefore more vulnerable to predation or to dislodgement by waves.

Here, we investigate whether *M. californianus* shells experienced changes in calcification from 1913 to 2010 in the southern portion of the California Current System (CCS: Point Conception to Baja California Sur). Previous studies found signs of *M. californianus* calcification stress in laboratory settings and historic records from northern sites (table 1). We hypothesize that there has been a long-term reduction in calcification, as indicated by thinner shells per unit length, a lower percentage of calcite-rich light growth banding, and increased crystallographic disorder. Alternatively, southern California mussels may exhibit signs of consistent or even increased calcification to cope with changing climate conditions, as recently found in the European congener *Mytilus edulis* [17]. We test these competing hypotheses to better understand how *M. californianus* is responding to environmental stressors at the southern end of its range and to determine whether there are regional differences in calcification sensitivity. The presence of coastal temperature records and an abundance of archived *M. californianus* twentieth to twenty-first century shell specimens from southern CCS sites provide a unique opportunity to compare environmental data and calcification metrics in an ecologically critical foundation species.

**Table 1.** Summary of findings from published literature on calcification patterns of *Mytilus californianus*.

reference	location	sample ages	finding	implications for calcification
Gaylord *et al.* [3]	Bodega Marine Laboratory, California	modern larval	larvae growing in high *p*CO_2_ resulted in smaller, thinner, weaker shells	projected reduction
Pfister *et al.* [14]; McCoy *et al.* [16]	Tatoosh Island, Sand Point, Washington	2000 BP, 1970s, 2010s	thinner shells in 2010s compared with 2000 BP and 1970s; increased crystallographic disorder in 2010s compared with 2000 BP and 1970s	reduction
Bullard *et al.* [23]	La Jolla, Corona del Mar, Avila Beach, California	1958, 1960, 2017, 2018	increased shell calcite relative to aragonite	increase
Vriesman *et al.* [24]	Bodega Bay, California	2002, 2003, 2019, 2020	thinner inner calcite layers and cross-sectional thicknesses relative to shell length, lowered contrast between dark–light growth bands in 2019–2020 relative to early 2000s	reduction

### (a) Study area

The southern portion of the CCS extends from Point Conception to the Baja California peninsula (figure 1). The southern CCS is influenced by the cold, south-flowing California Current, the warm north-flowing Southern California Countercurrent, and the Southern Californian Eddy [25–27]. The southern CCS is characterized by relatively weaker seasonal upwelling and warmer waters [26,27]. The southern CCS has experienced nearshore surface warming and OA over the past century, and multiple instrumental and proxy-based records indicate that warming has intensified over the second half of the twentieth century [28–31]. Increases in wind stress, upwelling strength, and anthropogenic carbon emissions have resulted in twentieth century acidification of southern CCS waters [31]. Centennial warming and acidification trends are compounded by interannual to decadal scale variability in temperature and upwelling strength due to the Pacific Decadal Oscillation (PDO) and El Niño Southern Oscillation (ENSO).

**Figure 1.**
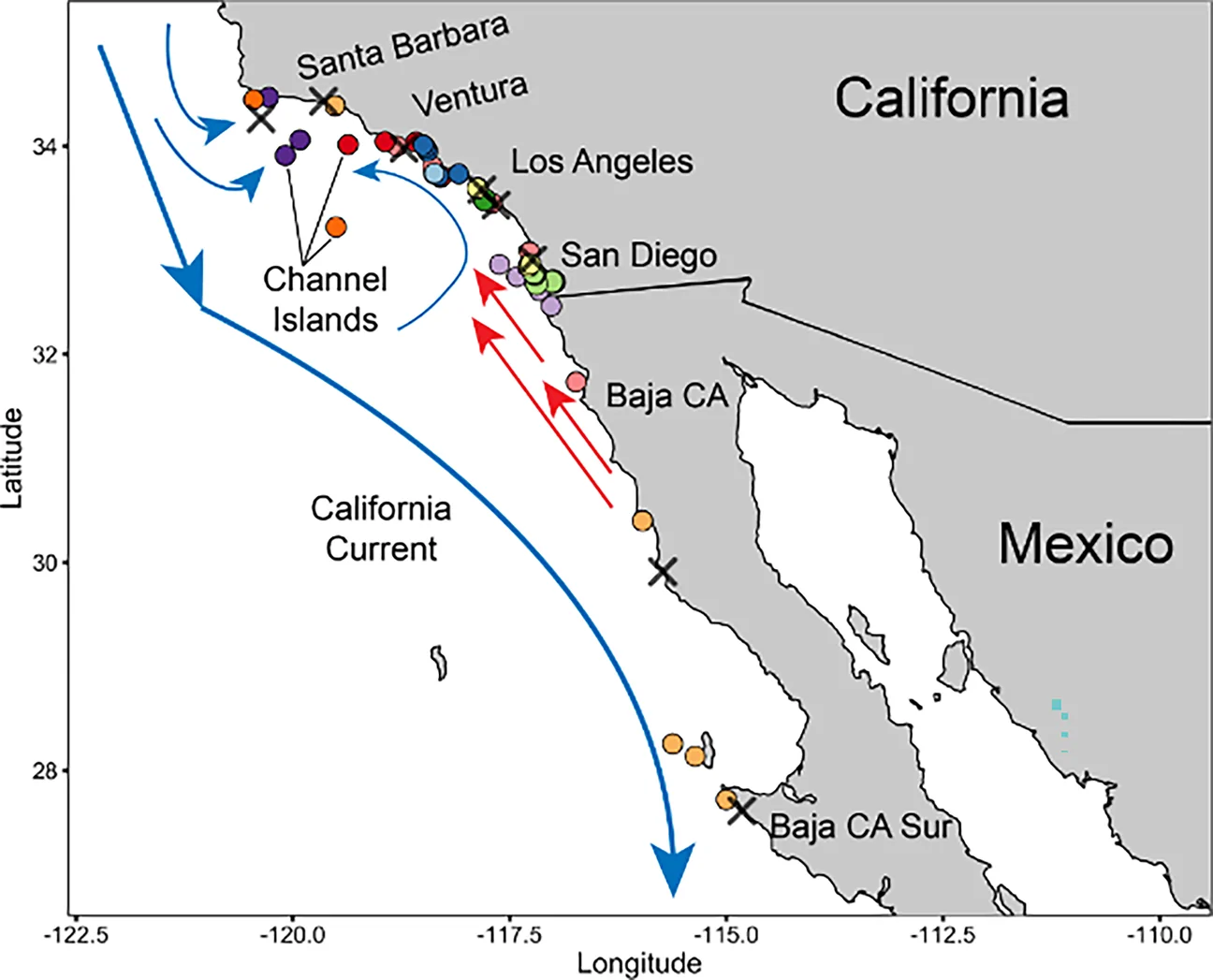
Map of *Mytilus californianus* collection locations. Blue arrows represent cold-water currents and red arrows represent warm-water currents. Each point colour denotes the decade of collection: 1910s—light blue; 1920s—dark blue; 1930s—light green; 1940s— dark green; 1950s—pink; 1960s—red; 1970s—light orange; 1980s—dark orange; 1990s—light purple; 2000s—dark purple; 2010s—yellow. Sea surface temperature record locations are denoted with X.

## 2. Material and methods

### (a) Instrumental observations

To contextualize shell characteristics within twentieth-century temperature patterns, we analysed multiple SST time series for southern CCS sites. We aimed to match the SST record and mussel records as closely as possible, although sites along the Baja California peninsula did not have continuous SST monitoring available. In these cases, we used annual temperature data points from intertidal loggers and long-term trends from the literature [32–34]. SST data locations and time series are shown in table 2.

**Table 2.** Summary of continuous sea surface temperature (SST) record locations and durations for the study area. Data from Baja California and Baja California Sur are excluded since these are not continuous records. Historic SST records were accessed from the University of California, San Diego Scripps Institute of Oceanography Shore Stations Program (available at https://shorestations.ucsd.edu/, last accessed 27 April 2022) and the National Oceanic and Atmospheric Administration's National Data Buoy Center (available at https://www.ndbc.noaa.gov/, last accessed 27 April 2022).

record name	location	record length	latitude (°N)	longitude (°W)
SIO - Scripps Pier	La Jolla (San Diego)	1916–2020	32.866198	117.254211
San Clemente	San Clemente	1965–2020	33.416193	117.618155
Newport Beach	Newport Beach	1924–2020	33.607255	117.929179
Point Dume	Point Dume	1957–2021	34.017639	118.826212
Santa Barbara	Santa Barbara	1955–2020	34.402714	119.692695
Station 46054	Santa Barbara Basin	2000–2005	34.272883	120.470103

No seawater *p*CO_2_ or pH time series data are available for this region throughout the full study period, but multiple studies have found that rising atmospheric CO_2_ levels have lowered surface ocean pH by up to 0.12 in the CCS since preindustrial times [35–37], and pH decline is markedly more severe for the southern CCS, with a regional pH decline of 0.21 since the year 1900 [31]. The longest record of direct pH observations in the Southern California Bight, the California Cooperative Oceanic Fisheries Investigation (CalCOFI) station 90.90, shows a clear acidification signal of −0.0015 ± 0.0001 yr^−1^ from 1984 to 2023 [38]. However, the station is hundreds of kilometres offshore and there are no similarly long-term records of pH closer to shore, so we sourced pH observations from the California Current Ecosystem buoys CCE1 and CCE2, located in the core of the California Current and on the shelf break, respectively, each with at least 10 years of data [39,40]. Finally, we sourced all Southern California Bight surface pH (less than 20 m depth) observations from the Multistressor Observations of Coastal Hypoxia and Acidification (MOCHA) synthesis, a nearshore-focused data compilation that includes oceanographic cruises, autonomous shore- and buoy-mounted sensors, and shore observations, with observations varying in location and frequency [41]. By examining observed rates of pH decline from the open ocean (CalCOFI 90.90) to nearshore waters (MOCHA), we aimed to contextualize the long-term acidification suggested by sediment cores [31] and bottle sampling [38], and to verify that the southern CCS OA signal was a persistent trend rather than a transient anomaly.

Historic SST data were normally distributed and analysed with parametric linear regression analysis to assess temporal trends, Welch's *t*-test to compare mean SSTs before and after 1970, and the *F*-test of equality of variances to compare SST variation before and after 1970. Simple linear regressions were performed on each pH data source to determine the rate of annual pH decline. To ensure that the continuous observational records of CCE1, CCE2, and MOCHA were not introducing seasonal bias relative to the CalCOFI 90.90 record, we performed linear regressions on the subset of pH observations that were taken within two weeks of a CalCOFI measurement.

### (b) Mussel shell specimens

We focus on the southern CCS owing to its well studied oceanography and the availability of archived *M. californianus* shell samples from this region. We accessed 130 dry-valve specimens of *M. californianus* spanning every decade from the 1910s to 2010. One fossil Pleistocene specimen (>11 700 years BP) is included as a comparison. Aside from the fossil, all samples were originally collected from intertidal environments south of Point Conception from the California counties of Santa Barbara, Ventura, Los Angeles, Orange and San Diego, and from the states of Baja California and Baja California Sur in Mexico (figure 1; supplementary material, table S1). Metadata included collection year, site name, latitude and longitude for every sample. While the geographical range is relatively broad (27.72° N to 34.47° N), this allowed us to obtain museum specimens from every decade over a century. We restricted our range to the southern portion of the CCS, which experiences the same upwelling regime and circulation patterns [27]. To disentangle geographical and temporal patterns, we divided the study area into four sub-regions: (1) Santa Barbara and Ventura, (2) Greater Los Angeles, (3) San Diego and Baja California, and (4) Baja California Sur.

### (c) Shell morphology

One intact valve from each of the 130 individuals was selected for non-destructive morphological measurements. Shell length was measured from umbo to commissure, and shell width was measured at the widest distance from the dorsal to ventral margin, using digital calipers (figure 2). The mass of each valve was measured to the nearest 0.1 g. A shell growth index was calculated for each specimen as the ratio between the log-transformed dry valve weight (g) and the log-transformed shell length (mm) [42].

**Figure 2.**
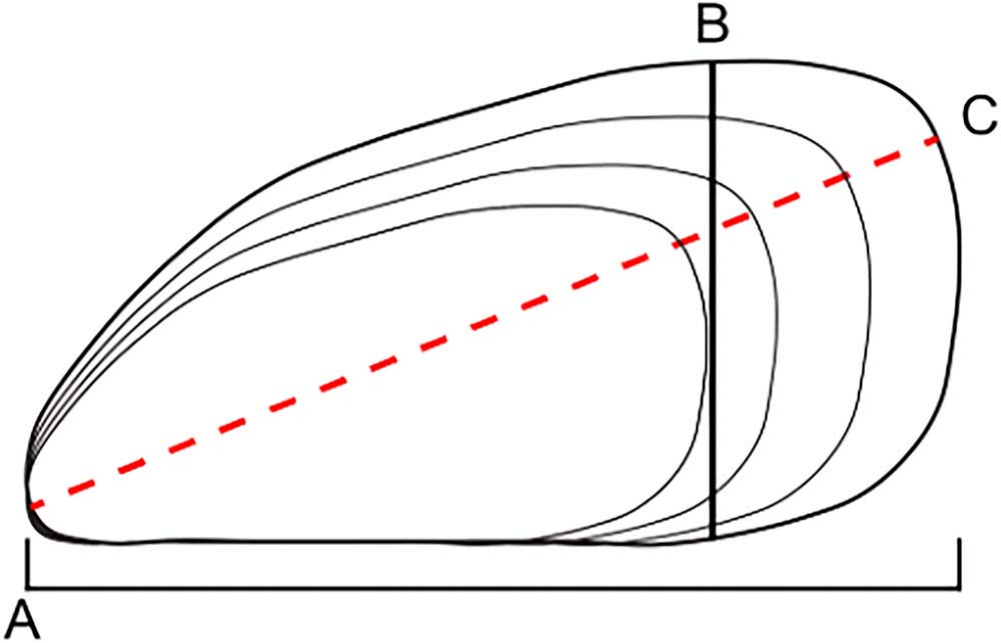
Diagram of valve measurement locations. (A) Line from anterior to posterior representing shell length parallel to hinge. (B) Line from dorsal to ventral showing shell width. (C) Red dashed line showing where shells were cut to produce a thin section along the axis of maximum growth.

### (d) Optical microscopy and statistical analyses

Thin sections were prepared from all specimens available for destructive analysis (*n* = 51). Valves were cut along the maximum growth axis (figure 2) using a low-speed saw with a 0.3 mm diamond wafering blade and polished to a thickness of 200 µm. Thin sections were examined and photographed using an Olympus transmitted light microscope and attached camera. The cross-sectional thickness of each shell was measured digitally at the thickest point adjacent to the umbo, where all mineralogical shell layers were present, and growth banding was visible [24]. Cross-sectional thicknesses were expressed relative to shell length and standardized as ratios in Excel. Photomicrographs of each specimen were converted to greyscale images to analyse growth banding in ImageJ. A transect perpendicular to shell length was drawn through the growth bands in the inner shell layer. Grey values were obtained along this transect to calculate the standardized grey-value variance for each specimen, used here as a proxy for the contrast between dark and light bands, or calcification consistency [24,43]. For each individual, grey-value pixels above the mean were considered light bands and grey-value pixels below the mean were considered dark bands. The percentage of light bands was calculated for each specimen (total light band amount (mm)/total dark band + light band amount (mm) × 100%). Standardized grey-value variances and the percentages of light bands were compared among all 51 thin-sectioned specimens.

The non-parametric Kruskal–Wallis rank sum test was used to assess whether there were significant spatial and temporal differences in shell characteristics with non-normal distributions or measurements with values less than or equal to zero, such as cross-sectional thickness, the percentage of light bands, and standardized grey-value variance. Analysis of variance (ANOVA) and Tukey's HSD test were used to compare log-transformed morphological characteristics (shell length and width) across collection years, decades and sub-regions. Spatial results (e.g. sub-regional trends) are secondary owing to uneven sampling distributions across sub-regions. Temporal trends in shell morphology were evaluated with multiple regression analysis (MRA), ordinary least squares regression (OLS), moving average analysis (MA), standardized major axis regression (SMA) and reduced major axis regression (RMA) since controls on shell growth are uncertain and highly variable and each regression model accounts for error differently. Note that different decades had a different number of shell samples available, which can skew statistical test results and regression model outputs. All statistical analyses were performed in R [44].

### (e) Exploratory electron backscatter diffraction observations

Three thin sections from the years 1913, 1956 and 2010 (*n* = 3 total) were chosen for preliminary electron backscatter diffraction (EBSD) following methodology from McCoy *et al.* [16]. EBSD scanning was performed using a Zeiss EVO-10 Variable Vacuum SEM at the University of California, Berkeley. Scanning electron microscopy (SEM) imaging was used to navigate towards the middle of the inner calcite layer adjacent to the shell's umbo to match the scanning sites for all three shells. Each thin section was analysed under low-vacuum mode with a 70° stage tilt to optimize Kikuchi patterns of biogenic calcium carbonate crystals [45]. Before each scan, diffraction patterns were indexed for calcite using EDAX OIM Analysis software to confirm that the confidence index was near 0.1, indicating high accuracy for crystallographic orientation data of each indexed mineral. The electron probe was operated at 10 nA, and the electron high tension was set to 20 kV.

## 3. Results

### (a) Observed temperature trends

Coastal SST measurements from the past century highlight nearshore surface warming in the southern CCS. There were significant increases in daily mean SSTs over time for all locations with continuous data (figure 3): linear regression, (1) La Jolla: *R*^2^ = 0.03, *F*_1,37046_ = 1033, *p* < 0.000, (2) Newport Beach: *R*^2^ = 0.004, *F*_1,34026_ = 131.9, *p* < 0.000, (3) Santa Barbara: *R*^2^ = 0.01, *F*_1,19933_ = 242.6, *p* < 0.000, (4) Point Dume: *R*^2^ = 0.001, *F*_1,22347_ = 17.39, *p* < 0.000, (5) San Clemente: *R*^2^ = 0.03, *F*_1,1984_ = 531, *p* < 0.000, (6) Station 46054: *R*^2^ = 0.01, *F*_1,2068_ = 18.84, *p* < 0.000).

**Figure 3.**
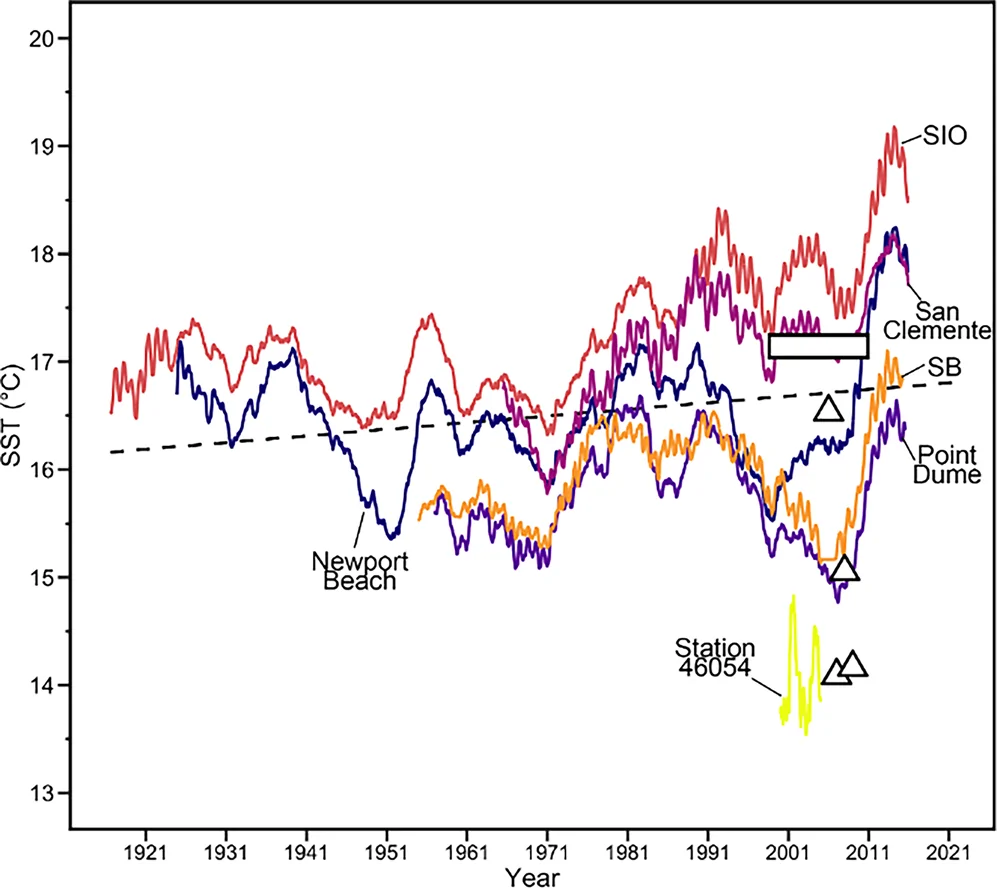
Sea surface temperature (SST) time series for each site. The short-term record (Station 46054, Santa Barbara Basin, in yellow) is plotted with a one-year running mean. Long-term records are plotted as a five-year running mean (La Jolla (SIO) SST in coral, Newport Beach SST in navy, San Clemente SST in magenta, Santa Barbara (SB) SST in orange, and Point Dume in purple). Triangles denote annual intertidal SST from loggers at Punta Baja, Baja California for 2006, 2007, 2009 and 2009 [34]. White bar denotes mean SST for 2000–2011 at Bahía Tortugas, Baja California Sur [32]. Black dashed line is the regression line for the La Jolla (SIO) temperature record.

All five long-term records (beginning in 1965 or earlier) show significantly higher mean annual temperatures after 1970 than before 1970 (table 3): Welch two-sample *t*-test, (1) La Jolla: *t* = 26.753, d.f. = 3672, *p* < 0.000, (2) Newport Beach: *t* = 7.4593, d.f. = 7153.2, *p* < 0.000, (3) Santa Barbara: *t* = 11.712, d.f. = 9481.2, *p* < 0.000), (4) Point Dume: *t* = 7.4593, d.f. = 7153.2, *p* < 0.000, and (5) San Clemente: *t* = 4.278, d.f. = 1995.2, *p* < 0.000.

**Table 3.** Summary statistics of each multi-year sea surface temperature (SST) record in the study area. All long-term records contain statistically significant increases in mean SST since 1970 (*p* < 0.000). *σ*, standard deviation. n.a., not applicable.

location	record length	min. (°C)	max. (°C)	mean (°C)	*σ*	pre-1970 mean (°C)	post-1970 mean (°C)
La Jolla (San Diego)	1916–2021	10.1	26.4	17.23	2.7	16.9	17.64
Newport Beach	1924–2020	9.9	25.2	16.5	2.4	16.4	16.6
Santa Barbara	1955–2020	9.9	23.4	15.93	2.3	15.63	16.04
Point Dume	1956–2020	8.9	23.9	15.77	2.3	15.56	15.82
San Clemente	1965–2020	9.8	26.2	17.14	2.6	16.89	17.12
Santa Barbara Basin	2000–2005	9.97	20.85	13.98	1.72	n.a.	n.a.

There were statistically significant increases in daily temperature variance since 1970 in all long-term records: *F*-test of equality of variances, (1) La Jolla: *F* = 1.079, d.f._num_ = 18018, d.f._denom_ = 19029, *p* < 0.000, (2) Newport Beach: *F* = 1.21, d.f._num_ = 18166, d.f._denom_ = 15861, *p* < 0.000, (3) Santa Barbara: *F* = 1.2661, d.f._num_ = 14961, d.f._denom_ = 4973, *p* < 0.000, (4) Point Dume: *F* = 1.3028, d.f._num_ = 18074, d.f._denom_ = 4274, *p* < 0.000, and (5) San Clemente: *F* = 1.126, d.f._num_ = 17845, d.f._denom_ = 1640, *p* = 0.001. While the significant increase in mean annual temperature for all sites is evidence of sustained warming, we also found an increase in daily maximum temperatures for Newport Beach and La Jolla (supplementary material, figure S1). When summer SSTs (June–August) were seasonally averaged, conditions at La Jolla and Newport Beach were warmer over both time series, with steeper summer warming trends post-1970 for both of these sites (supplementary material, figures S2 and S3).

### (b) Inferred pH trends

In the absence of continuous nearshore pH data over the study period, we referenced OA trends from offshore CCS sediment cores (1895–2000) [31] and bottle samples (1984–2023) [38]. We analysed coastal pH data from 2010–2022 and found that pH declined across all four records (CalCOFI, MOCHA, buoys CCE1 and CCE2; supplementary material, figure S4), with acidification rates steepening closer to shore (supplementary material, figure S5). We found that pH at the CalCOFI 90.90 station is declining at −0.0015 ± 0.0004 yr^−1^, in agreement with Wolfe *et al.* [38]. We note that the discrepancy between our standard error and their reported standard error of 0.0001 likely reflects that Wolfe *et al.* de-seasoned the CalCOFI observations while we did not. At CCE1, we found a rate of −0.0086 ± 0.0001 yr^−1^, which was close to the rate we found at CCE2 (−0.0079 ± 0.0001 yr^−1^). Finally, the nearshore-skewed MOCHA observations had a rate of pH decline of −0.011 ± 0.0002 yr^−1^. When analysing the subset of CCE1, CCE2 and MOCHA observations that corresponded with CalCOFI 90.90 sampling times, the calculated pH slopes were similar and identically ordered, but CCE1 had a larger gap, and CCE2 had a rate of decline similar to that of MOCHA. The pH data analysed here do not overlap with the full study period and are therefore used to infer rather than identify drivers of any observed changes in calcification.

### (c) Shell characteristics: temporal patterns

Non-destructive morphological measurements (*n* = 130 specimens) revealed that shell shape did not change significantly over time. Shell width grew proportionally to length across all samples (slope = 0.91), but there was no significant change in shell shape since 1913 (figure 4; multiple regression analysis using year and sub-region as factors, *x* = log(Length), *y* = log(Width): *F*_5,123_ = 533.9, *p* < 0.000; supplementary material, table S2). When sorted by decade, RMA regression revealed highly consistent slopes (width-to-length ratios) through time in decades with three or more samples (supplementary material, table S3). All regression models found log-transformed shell width to length relationships with slopes above 0.9, revealing proportional growth as mussels age ontogenetically and through time ( table 4; supplementary material, figure S6).

**Figure 4.**
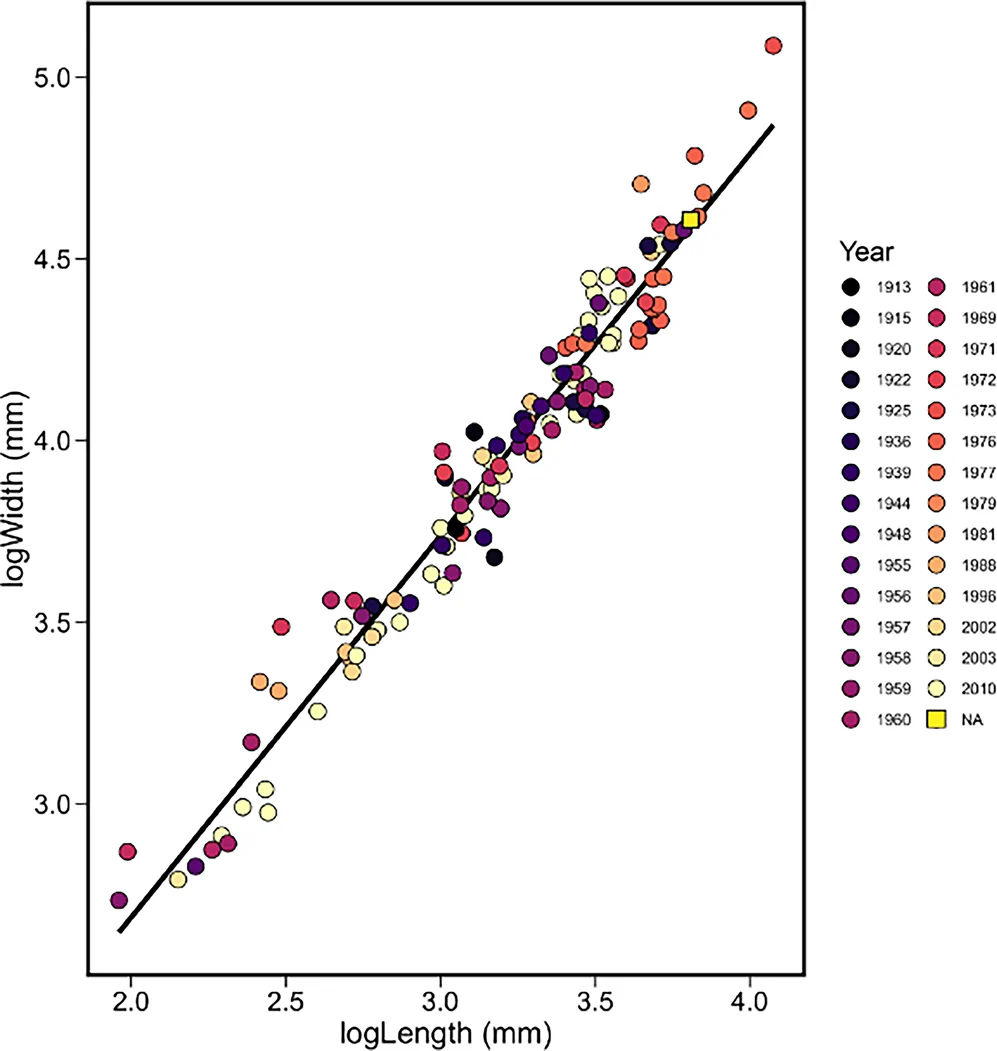
Relationships between log-transformed shell width and log-transformed shell length. Each point is for a unique specimen (*n* = 130), and each colour represents a different year (see legend). The regression line represents the trend for all specimens. NA is the fossil specimen.

**Table 4.** Results from all regression analysis methods: ordinary least squares (OLS), moving average (MA), standardized major axis (SMA), and reduced major axis (RMA).

method	*y*-intercept (*b*)	slope (*m*)	angle (degrees)
OLS	−0.373	0.905	42.146
MA	−0.457	0.926	42.809
SMA	−0.464	0.928	42.863
RMA	−0.478	0.932	42.969

Destructive analyses (*n* = 51 specimens) found no significant changes in quantitative shell characteristics through time (figure 5; supplementary material, table S5). There was no significant relationship between standardized cross-sectional thickness per shell length and decade or between standardized cross-sectional thickness and sub-region (Kruskal–Wallis rank sum test: *p* > 0.8 for decade; *p* > 0.5 for sub-region). There was no significant change in the light band percentage through time (Kruskal–Wallis rank sum test: *p* = 0.39 with year as the factor; *p* = 0.51 with decade as the factor). Lastly, the standardized grey-value variance, a proxy for growth band contrast, did not change significantly over time (Kruskal–Wallis rank sum test: *p* = 0.93 with year as the factor; *p* = 0.78 with decade as the factor).

**Figure 5.**
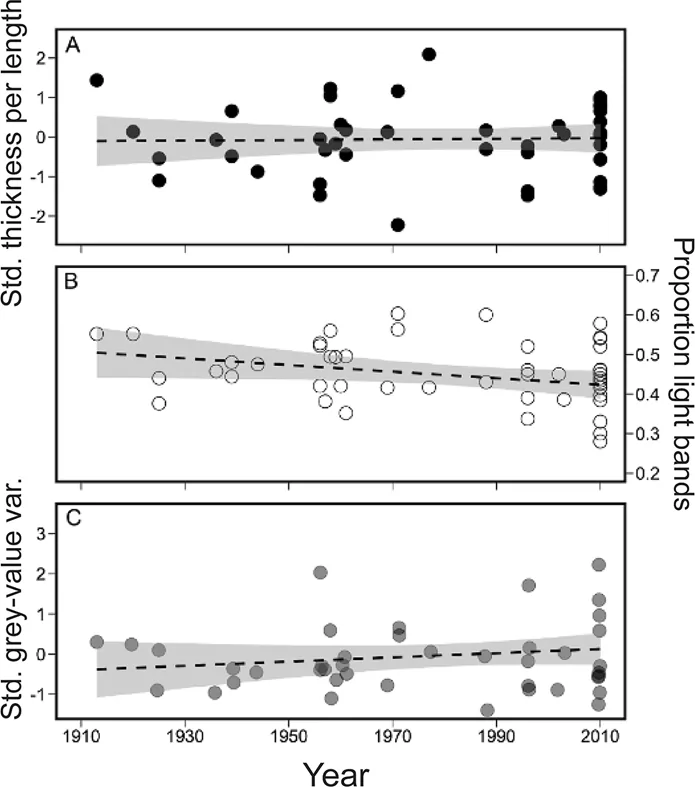
Quantitative *M. californianus* shell characteristics over time, including standardized cross-sectional thickness per shell length (A), proportion of light bands in the inner calcite layer (B) and standardized grey-value variance of dark–light banding (C). Linear regressions are plotted for each shell characteristic with a 95% confidence interval, although none of these shows statistically significant trends.

All 51 specimens, including the fossil shell (supplementary material, figure S9), contained an inner prismatic calcite layer, a middle layer of aragonite, and an outer layer of calcite. There were no visually apparent temporal changes in mineralogical layering in thin sections (supplementary material, figure S10).

### (d) Shell characteristics: geographical patterns

Non-destructive measurements (*n* = 130 specimens) found some morphological differences when divided into geographical sub-regions; there was a statistically significant difference in shell morphology through time as measured by a reduction in log(Width (mm))/log(Length (mm)) for two of the sub-regions: multiple linear regression: (i) Greater Los Angeles, *p* = 0.048 and (ii) San Diego and Baja California, *p* = 0.002; supplementary material, table S2 and figure S7.

Linear regression on shell growth indices (log(Mass (g))/log(Length (mm))) found a statistically insignificant slope of −0.001382. Growth indices did not vary significantly across sub-regions (*p* = 0.2; figure 6). Shell growth indices were variable through time, but the late 1970s was a period with high growth indices for shells from all four sub-regions, and particularly for Santa Barbara and Ventura and San Diego and Baja California shells, although sample sizes were uneven across groups. There was no change in shell size over time, as measured by standardizing shell length relative to valve mass. Samples from the 1970s had the highest calcification indices and the largest shells, although there was only one sample from Greater Los Angeles collected in the 1970s (figure 6; supplementary material, figure S8).

**Figure 6.**
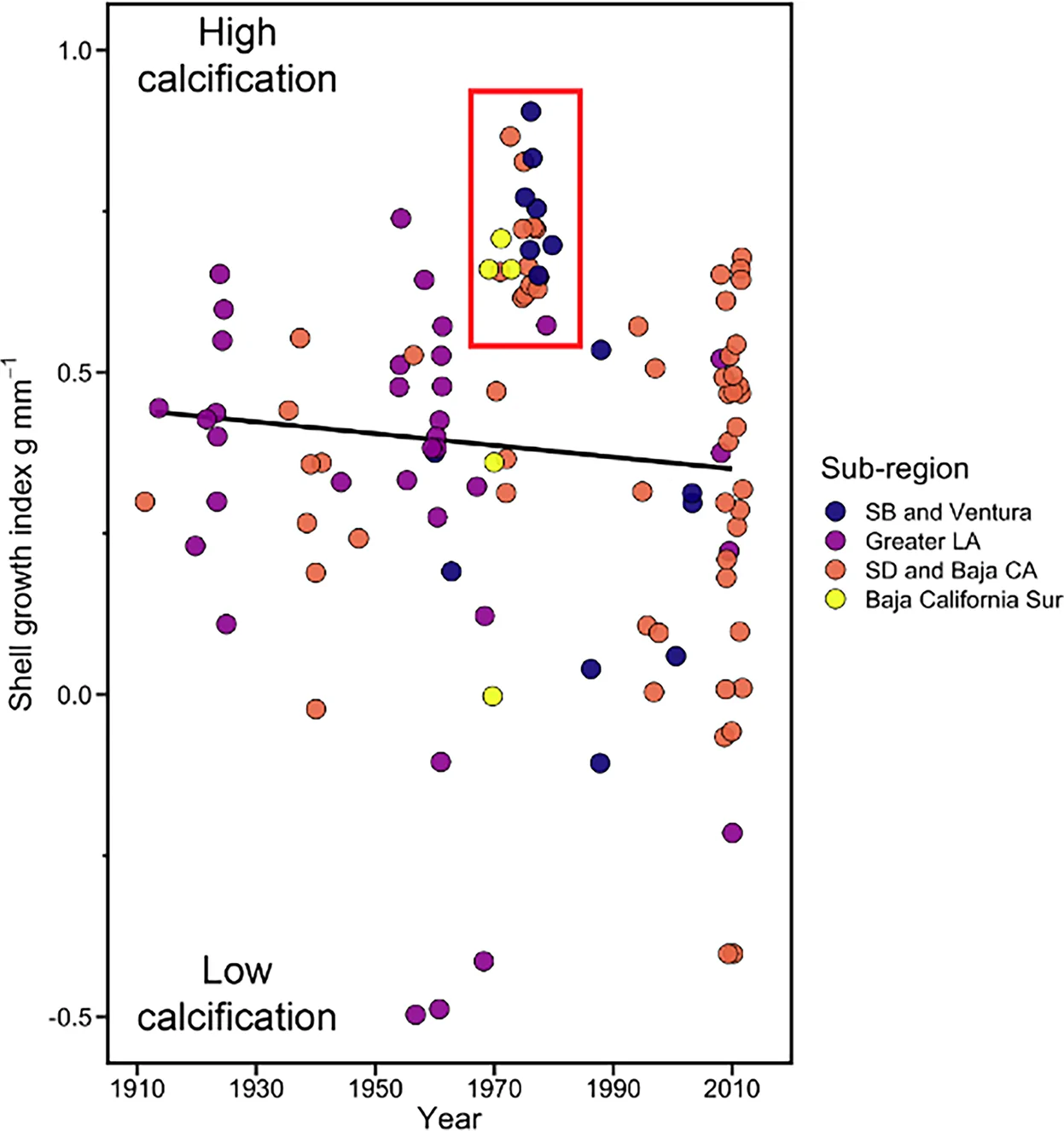
Shell growth indices of all specimens through time sorted by sub-region (see legend): Santa Barbara (SB) and Ventura (navy), Greater Los Angeles (Greater LA) (magenta), San Diego (SD) and Baja California (Baja CA) (coral), Baja California Sur (yellow). Black line is plotted for all specimens and represents a non-significant slope of −0.001382, *p* = 0.2. Red box highlights a period of higher growth indices.

There was a statistically significant relationship between sub-region and shell shape when the temporal component was excluded (ANOVA: shell width/length with sub-region as the only factor, *p* = 0.0033). Shells from San Diego and Baja California had higher width to length ratios than shells from Santa Barbara and Ventura (Tukey HSD: *p* = 0.03) and shells from Baja California Sur had lower width to length ratios than shells from San Diego and Baja California (Tukey HSD: *p* = 0.04).

Destructive analyses found no further differences between shell characteristics related to sub-region. Standardized cross-sectional thickness per shell length did not significantly differ among sub-regions (ANOVA: *p* > 0.285). Similarly, the percentage of light bands and the standardized grey-value variance did not vary by sub-region (Kruskal–Wallis rank sum test: percentage of light bands, *p* = 0.133; standardized grey-value variance, *p* = 0.43). Shell characteristics by sub-region are summarized in supplementary material, table S4.

### (e) Preliminary crystallographic observations

EBSD maps revealed the crystallographic orientations and crystal size within the inner calcite layer of three *M. californianus* specimens (figure 7). The 1913 shell contained well organized microstructure in its inner calcite layer; the elongate calcite crystals were similar in size and formed needle-like crystals characteristic of biogenic prismatic calcite [46,47]. Dark areas in the crystallographic map represent the absence of crystal backscatter in organic-rich dark bands. There was some variability in crystallographic orientation (figure 7A) despite highly uniform crystal sizes. The 1956 shell also contained large, similar crystal sizes throughout the scan, and crystallographic orientations were uniform (figure 7B). Lastly, the 2010 shell contained a greater variety of crystallographic orientations, more variability in crystal sizes, and smaller crystals (figure 7C).

**Figure 7.**
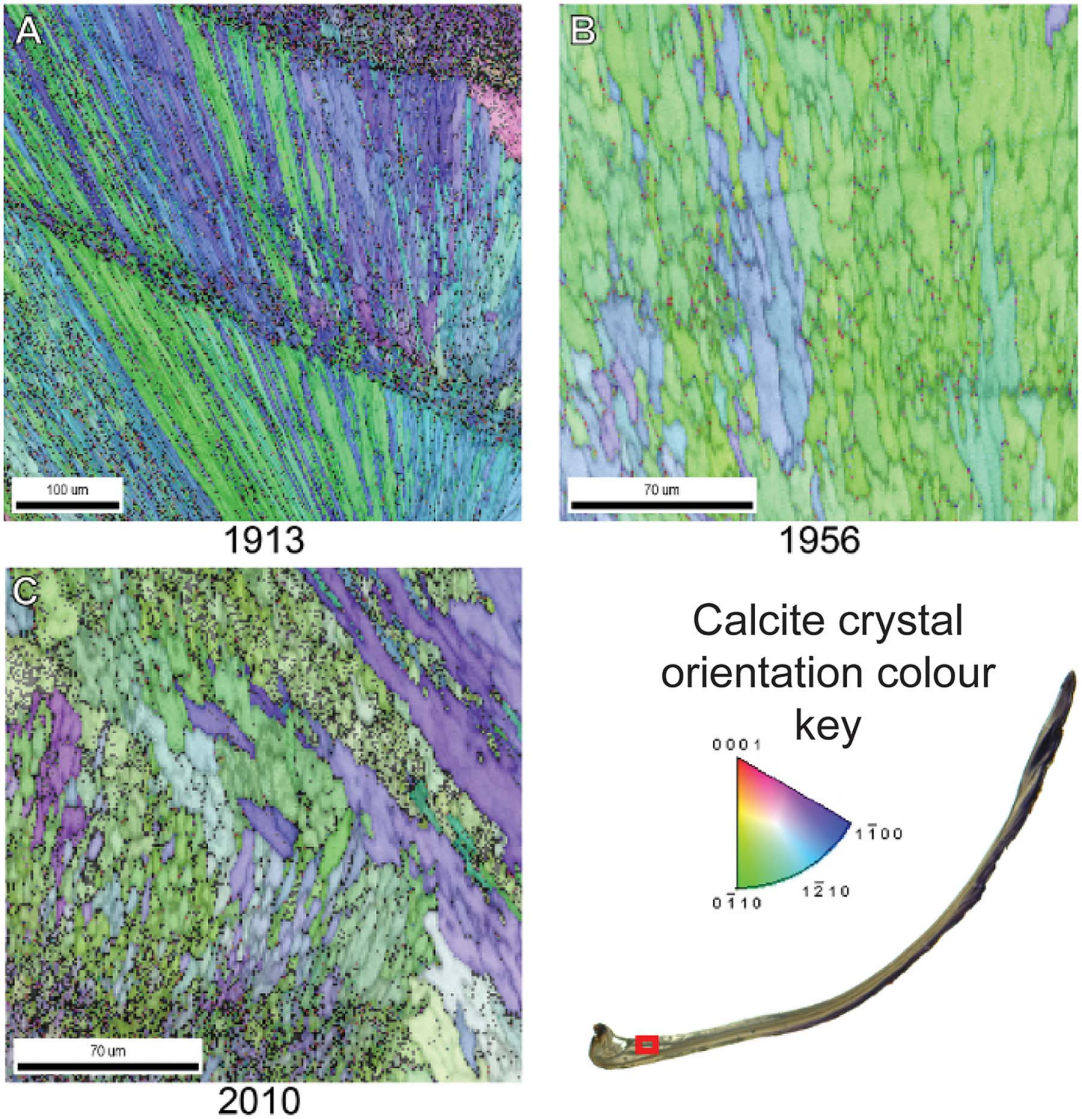
Electron backscatter diffraction (EBSD) scans in the inner calcite layer of three individuals: 1913, La Jolla (A); 1956, San Diego (B); and 2010, Point Dume (C). Note that the scale bars represent 100 µm for (A) and 70 µm for (B) and (C). The youngest shell material is at the top of each scan. The aragonite layer is below the field of view. Crystallographic orientations are shown relative to the (0001) plane, as shown in the crystal orientation key. The red box in the shell cross-section is the EBSD scan location. Scale bars: (A) 100 µm; (B,C) 70 µm.

## 4. Discussion

We hypothesized that a shift toward significantly warmer and more acidic conditions in the southern CCS would result in a marked shift in calcification patterns in *M. californianus.* However, *M. californianus* shells showed signs of remarkably stable calcification patterns. None of the quantitative shell characteristics measured here changed significantly over this century-long period even though all long-term SST records in southern California revealed significant overall warming. Maximum temperatures increased while minimum temperatures did not, meaning that there was a broadening of the annual temperature range rather than a discrete shift.

In addition to warming, acidification has also occurred in the CCS since the preindustrial era [35,36]. Based on calculated acidification rates, we can infer that significant pH declines—such as the 0.21 suggested by Osborne *et al.* [31]—are plausible in the region and perhaps even more severe in nearshore waters. The rates of pH decline we observed in CCE1 and CCE2, and in the MOCHA dataset far exceed those expected from equilibrium between ocean–atmospheric CO_2_ concentrations. We do not suggest that our 10-year observational record is representative of the last century, but it does show that large declines in the region are possible, even on relatively short timescales, and have potentially been underestimated from offshore records (e.g. core samples), especially since coastal upwelling intensifies acidification [48]. For example, in the nearshore-skewed MOCHA dataset, the pH rate of decline of −0.011 ± 0.02 yr^−1^ exceeds the rate of pH decline found by Osborne *et al.* [31] since the pre-industrial era, indicating that rapid short-term declines are possible in nearshore environments. We found that OA in coastal southern California continued to intensify from 2010 to 2023 (supplementary material, figure S4) and that acidification rates are steeper closer to shore (supplementary material, figure S5), indicating that intertidal mussels may experience more extreme OA conditions than biota farther offshore.

Unlike *M. californianus* shells from Washington and northern California [14,16,24], mussels growing in the southern CCS exhibited calcification consistency throughout the twentieth century, except for a subset of higher calcification indices in late-1970s specimens (figure 6), coeval with a warm shift in the CCS [30]. It is possible that mussels may have precipitated more shell material during this brief period of substantial warming (figures 3–6), although a larger sample size and analysis of historic nutrient records and food availability would help to validate this trend. Any potential relationship between elevated shell growth indices and high 1970s temperatures is different from documented shell growth–SST relationships at a northern California site, where mussels preferentially calcified during moderately cool conditions (approx. 13°C) [24]. However, southern California mussels have been reported to calcify more rapidly in warmer conditions (15 to 19°C) [20], suggesting regional acclimatization. Our results do not contradict the positive relationship between faster growth and higher temperatures for southern populations of *M. californianus*, as long as temperatures do not exceed their thermal calcification threshold (approx. 26°C) [49].

Geographical differences in the CCS upwelling regime cause the southern CCS to experience warmer conditions than the northern California coast, so southern populations of *M. californianus* may be less sensitive to anthropogenic warming, and in response, calcification patterns have remained stable. Similarly, mussels calcifying in high-intensity upwelling in northern California may be more acclimatized to acidifying conditions, since upwelling-induced low pH is a natural, seasonally occurring phenomenon in this region. Local, population-specific acclimatization would also contribute to the OA-related shell thinning and heightened crystallographic disorder found in Washington *M. californianus* shells; Tatoosh Island and Sand Point, Washington experience significantly less upwelling relative to the northern California coast, so Washington mussels were perhaps poorly adapted to low pH and more susceptible to long-term, anthropogenic OA trends [14,16,24,50,51].

Although 1970s warming aligned with a brief window of high shell growth indices, there was no indication that mussel shell growth increased as warming progressed temporally in the CCS, which is an additional line of evidence for a regional disparity in shell growth patterns. Prior studies have identified Point Conception as an ecological boundary [52–54]. It is possible that Point Conception may also function as a calcification transition zone separating cool-water acclimatized northern mussel populations and warm-water acclimatized southern mussel populations.

Preliminary EBSD data from three shells collected in 1913, 1956 and 2010 present the only evidence for differences in shell characteristics through time. Among the three specimens, EBSD revealed greater crystallographic variability in 2010 than in 1913 and 1956 (figure 7). The 2010 shell exhibited the widest range of crystal sizes (figure 7C), while the earliest twentieth-century shell (1913) exhibited uniform crystal sizes. All three shells showed some variability in crystallographic orientations, as expected in biogenic calcite [16]. Since no quantitative shell characteristics changed as warming occurred in the CCS, it is possible that declining pH over the study period [31,38] is responsible for the crystallographic disorder observed here, although it is important to note that EBSD observations are preliminary and only based on three individuals. Further EBSD analysis of a larger sample size of twenty-first-century shells is required to confirm the timing and extent of the crystallographic shift, if present. If crystallographic control has indeed declined in southern *M. californianus* shells, it is unclear whether this is a deleterious effect or an adaptive coping mechanism. Reduced control of crystallography could be a sign of hindered calcification, or it could be a way to calcify more quickly in response to suboptimal conditions. It is therefore unknown if a shift in microstructural properties will have significant implications for other aspects of *M. californianus*’ life history and the ecosystems it supports. Increased crystallographic disorder could compromise shell structural integrity, making organisms more vulnerable to changing environments and increased predation pressure [55,56]. In the calcitic shell of the oyster *Crassostrea virginica*, reduced hardness was associated with increased crystallographic disorientation when oysters were cultured under OA scenarios up to 1000 µatm *p*CO_2_ [55]. In the mussel *M. edulis*, similar laboratory OA conditions resulted in a loss of crystallographic control, which may hinder shell growth or repair and impede the shell's protective function [56]. If predation pressure escalates as environmental changes intensify, crystallographic disorganization and a corresponding decline in shell hardness will compound mussel vulnerability.

High variability among our results suggests that factors not analysed here, including micro-habitat (e.g. tidal position, thickness of mussel bed) and ontogenetic age, may also influence responses to a changing climate across a population [57]. Slower calcification has been tied to both ontogenetic growth and tidal height for *M. californianus* [58–60]. Variability introduced by micro-scale biological, ecological and spatial conditions may dominate over broader trends when examining population-specific calcification patterns. It is possible that long-term trends could emerge if we could analyse the same shell characteristics in an ideal suite of samples with known and uniform ontogenetic ages and tidal positions through time, although this is not possible with existing archival samples owing to limited specimens, destructive sampling protocols and inconsistent metadata across collections.

Although unexpected, our results are consistent with other signs of resilience found in the *M. californianus* species and within the *Mytilus* genus (e.g. [17,23]). Other cases of calcification stability have also been documented in other marine calcifying phyla from temperate regions; in the calcitic brachiopod *Calloria inconspicua*, shell morphology, thickness, and elemental composition were constant over the past 120 years despite ocean warming and higher *p*CO_2_ over decadal scales in New Zealand [15].

## 5. Conclusions

We found minimal evidence of shifting calcification patterns in *M. californianus* despite observed secular warming and inferred OA in the southern CCS over the twentieth century. We interpret the unchanging shell characteristics as evidence of calcification resilience in *M. californianus* populations growing at the southern end of their range. In contrast, California mussel populations growing in cooler waters farther north (e.g. northern California to Washington) have shown signs of calcification stress over this same period. Latitudinally disparate calcification responses in *M. californianus* suggest that northern populations may be more vulnerable to ocean warming, and conversely, southern populations that do not commonly experience upwelling-induced low-pH conditions may be more vulnerable to projected anthropogenic OA conditions rather than warming.

Although we found no evidence of calcification stress from quantitative metrics, we did observe crystallographic disorder in a single shell from 2010, but further analysis of recent shells is required to confirm whether a crystallographic shift has indeed occurred, and if so, whether it is detrimental to *M. californianus* and the ecosystem it supports. Understanding the ways in which this critical foundation species will respond to climate change across a variety of spatial and temporal scales is valuable for the hundreds of species that rely on *M. californianus* for habitat and protection, and consequently, for the ecosystems and human communities that benefit from coastal environments.

## Supplementary Material



## Data Availability

Data and code for this article are available at [61]. Supplementary figures and tables are provided in the electronic supplementary material, available online [62].
