# Supplementary material for: Calcification patterns of a foundation species reveal resilience to environmental change in southern California over the twentieth century
Source: Proc Biol Sci. 2026 Oct 21;293(2081):20261964. doi: 10.1098/rspb.2026.1964 (PMC13682939; doi:10.1098/rspb.2026.1964)
Supplement: Supplementary file 1 [file rspb.2026.1964.zip › rspb.2026.1964/rspb20261964_si_001.docx]

**SUPPLEMENTARY INFORMATION** for “Calcification patterns of a foundation mussel species reveal resistance to environmental changes in the southern California Current System over the 20^th^ century”

**Table S1.** Summary of specimens (n = 130) used in this study and their sources, locations, and collection years.

| Sample Source (individual or institution name) | Collection Localities | Collection Years |
| --- | --- | --- |
| Elizabeth Bullard, UC San Diego | La Jolla Tide Pools, Corona del Mar, SIO Pier Pilings, Dike Rock Tide Pools | 1996, 2010 |
| California Academy of Sciences, Invertebrate Zoology | Santa Monica Beach, Cabrillo Beach, Point Fermin, Anacapa Island, La Jolla, Dutch Harbor San Nicolas Island, Alegria, Fraser Point Santa Cruz Island, Johnsons Lee Santa Rosa Island, | 1925, 1960, 1961, 1973, 1981, 2002, 2003 |
| Natural History Museum of Los Angeles County, Malacology | Portuguese Bend Palos Verdes Peninsula, Venice Beach, White Point Palos Verdes Peninsula, Newport Beach Jetty, Carpinteria State Beach, Government Point, E Punta Azufre Baja California, Rocas Pináculo Islas San Benito Baja California, Piedra Colorada Isla Cedros, Punta Rompiente Baja California Sur | 1915, 1922, 1925, 1969, 1971, 1972, 1976, 1977, 1979, 1988 |
| Santa Barbara Museum of Natural History, Invertebrate Zoology | La Jolla, Playa del Rey, Anaheim Landing, North Island Coronado Jetty, South Laguna Beach, Point Dume, Palos Verdes, Whites Point, Corona del Mar, Point Fermin, Newport Mesa | 1913, 1920, 1936, 1944, 1956, 1957, 1959, 1960 |
| UC Museum of Paleontology, Invertebrates | Mission Bay, San Diego, Del Mar, Punta Banda, Dana Point, Solana Beach, Cabrillo Beach, Topanga Beach, Venice Beach | 1939, 1948, 1958, 1960, 1961 |

**Figure S1.**

Annual maximum daily SST plotted for all available SST time series in the study area. La Jolla (SIO) SST is in coral, Newport Beach SST is in navy, San Clemente SST is in magenta, Santa Barbara (SB) SST is in orange, Station 46054 (Santa Barbara Basin) is in yellow, and Point Dume is in purple (all labeled on plot).

**Figure S2.**

La Jolla (SIO) summer SST over time. Each point is a seasonally averaged summer mean SST (June to August, n=106), where gray points represent observations prior to 1970 and black points represent observations after 1970. The horizontal dotted line demarcates 1970. Regression lines represent the trends for each time period. (Prior to 1970: y = -4.814*10^-5^x+1.925 (R^2^=0.166, p=0.002), after 1970: y = 9.955*10^-5^x+19.43(R^2^=0.402, p=4.523*10^-7^)).

**Figure S3.**

Newport Beach summer SST over time. Each point is a seasonally averaged summer mean SST (June to August, n=96), where gray points represent observations prior to 1970 and black points represent observations after 1970. The horizontal dotted line demarcates 1970. Regression lines represent the trends for each time period. (Prior to 1970: y = -5.209*10^-5^x+18.11(R^2^=0.120, p=0.02), after 1970: y = 8.039*10^-5^x+17.99 (R^2^=0.237, p<0.001)).

**Figure S4.**

Available southern California pH time series data spanning 2010-2022 (CalCOFI) and Kennedy et al. (2023). Legend lists data source according to distance offshore, ranging from farthest offshore (CalCOFI 90.90) to closest to shore (MOCHA). MOCHA includes all pH observations from Kennedy et al. (2023) excluding moorings CCE1 and CCE2.

**Figure S5.**

Acidification rates (expressed as yearly slope of pH change) plotted against distance from shore for 2010-2022. “All” represents all aggregated available pH data (Kennedy et al., 2023) and “Slim” represents pH data filtered to within two weeks of a CalCOFI 90.90 measurement to temporally sync datasets.

**Table S2.** Coefficients of multiple regression analysis using Year and Sub-Region as factors, x = log(Length), y = log(Width): *F*_5,123_ = 533.9, *p* < .000) for shell width vs. shell length. Includes all 130 shells.

| Independent (x) | Dependent (y) | slope (*m*) | SE | y-intercept (*b*) | *p*-value | R^2^ |
| --- | --- | --- | --- | --- | --- | --- |
| log(length) mm | log(width) mm | 0.91 | 0.68 | 0.06 | < .000 | 0.96 |

**Table S3.** Slopes of RMA regression lines for shell width to length ratios for each decade.

| Decade | slope (*m*) | number of shells | r^2^ |
| --- | --- | --- | --- |
| 1910 | 0.22 | 2 | 1 |
| 1920 | 0.97 | 9 | 0.84 |
| 1930 | 0.85 | 9 | 0.85 |
| 1940 | 0.87 | 2 | 1 |
| 1950 | 0.94 | 13 | 0.97 |
| 1960 | 1.1 | 16 | 0.94 |
| 1970 | 0.98 | 27 | 0.9 |
| 1980 | 0.93 | 4 | 0.97 |
| 1990 | 0.89 | 6 | 0.99 |
| 2000 | 0.88 | 7 | 0.97 |
| 2010 | 0.84 | 34 | 0.98 |

**Table S4.** Summary statistics of destructive analyses for each quantitative shell characteristic sorted by sub-region. Mean denoted with x̄ and standard deviation denoted with σ.

| Sub-  region | *n* (whole valves) | x̄ shell growth index | σ shell growth index | *n* (thin sections) | x̄ std. cross-  sectional thickness per length | σ std. cross-  sectional thickness per length | x̄ std. gray-value variance | σ std. gray-value variance | x̄ portion of light bands | σ portion of light bands |
| --- | --- | --- | --- | --- | --- | --- | --- | --- | --- | --- |
| Santa Barbara and Ventura | 19 | 0.47 | 0.32 | 6 | 0.41 | 0.85 | -0.46 | 0.59 | 0.44 | 0.09 |
| Greater Los Angeles | 38 | 0.28 | 0.37 | 16 | -0.42 | 0.64 | -0.12 | 0.86 | 0.44 | 0.12 |
| San Diego and Baja California | 68 | 0.37 | 0.3 | 27 | 0.21 | 1.06 | 0.13 | 1.15 | 0.44 | 0.07 |
| Baja California Sur | 5 | 0.48 | 0.3 | 2 | -0.53 | 2.39 | 0.56 | 0.13 | 0.58 | 0.03 |

**Table S5.** Summary statistics of destructive analyses for each quantitative shell characteristic sorted by decade. Mean denoted with x̄ and standard deviation denoted with σ. Shell growth index is log(valve mass (g))/log(shell length (mm)).

| Decade | *n* (whole valves) | x̄ shell growth index | σ shell growth index | *n* (thin sections) | x̄ std. cross-sectional thickness per length | σ std. cross-sectional thickness per length | x̄ std. gray-value variance | σ std. gray-value variance | x̄ portion of light bands | σ portion of light bands |
| --- | --- | --- | --- | --- | --- | --- | --- | --- | --- | --- |
| 1910 | 2 | 0.38 | 0.1 | 1 | 1.43 | NA | 0.3 | NA | 0.55 | NA |
| 1920 | 9 | 0.41 | 0.17 | 3 | -0.5 | 0.62 | -0.19 | 0.62 | 0.46 | 0.09 |
| 1930 | 9 | 0.3 | 0.19 | 3 | 0.03 | 0.58 | -0.68 | 0.3 | 0.46 | 0.02 |
| 1940 | 2 | 0.29 | 0.06 | 1 | -0.88 | NA | -0.46 | NA | 0.48 | NA |
| 1950 | 13 | 0.34 | 0.39 | 7 | -0.13 | 1.01 | -0.03 | 1.04 | 0.49 | 0.06 |
| 1960 | 16 | 0.2 | 0.39 | 4 | 0.04 | 0.33 | -0.41 | 0.3 | 0.42 | 0.06 |
| 1970 | 27 | 0.2 | 0.19 | 3 | 0.34 | 2.27 | 0.4 | 0.31 | 0.53 | 0.1 |
| 1980 | 4 | 0.63 | 0.39 | 2 | -0.07 | 0.33 | -0.73 | 0.95 | 0.52 | 0.12 |
| 1990 | 6 | 0.29 | 0.28 | 5 | -0.02 | 1.97 | 0.002 | 1.05 | 0.43 | 0.07 |
| 2000 | 7 | 0.27 | 0.15 | 2 | 0.17 | 0.14 | -0.43 | 0.65 | 0.42 | 0.05 |
| 2010 | 34 | 0.22 | 0.35 | 19 | 0.08 | 0.77 | 0.24 | 1.28 | 0.42 | 0.11 |
| Fossil | 1 | 0.25 | NA | 1 | -0.88 | NA | 1.13 | NA | 0.43 | NA |

**Figure S6.**

RMA analysis shows proportional growth (*m* = 0.93) between shell length and width for all specimens. Each point represents a single specimen, and colors represent different decades (see legend). Red line represents the RMA regression line (*m* = 0.93, *b* = -0.48, angle = 42.97°) and gray lines indicate the confidence limits.

**Figure S7.**

Relationships between log-transformed shell width and log-transformed shell length for all specimens. Each point is a unique specimen, and each color represents a different sub-region of the study area (see legend). Simple linear regression line (*m* = 0.91) represents the trend for all specimens, as in Figure 4.

**Figure S8.**

Shell size over time, expressed as standardized shell length relative to mass.

**Figure S9.**

Cross-sectional view of the Pleistocene *M. californianus* specimen showing the presence of the inner calcite layer. Growth direction of the inner calcite layer is indicated by the white arrow. The youngest shell material is towards the top of the image. The shell exterior is towards the bottom of the image.

**Figure S10.**

Photomicrographs of four thin-sectioned specimens collected from La Jolla in 1913 (A), Point Dume in 1956 (B), San Diego in 2010 (C), and another specimen from San Diego in 2010 (D). The aragonite and outer calcite layers are also visible, except for in (B), where the aragonite layer begins just to the left of the field of view. Each photo was taken adjacent to the umbo and focuses on the inner calcite layer with dark-light growth banding. The inner calcite layer grows inward as indicated by the white arrows.
